# The reproductive status determines tolerance and resistance to *Mycobacterium marinum* in *Drosophila melanogaster*

**DOI:** 10.1093/emph/eoad029

**Published:** 2023-09-06

**Authors:** Marta Arch, Maria Vidal, Esther Fuentes, Anmaw Shite Abat, Pere-Joan Cardona

**Affiliations:** Tuberculosis Research Unit, Germans Trias i Pujol Research Institute (IGTP), Badalona, Catalonia, Spain; Comparative Medicine and Bioimage Centre of Catalonia (CMCiB), Germans Trias i Pujol Research Institute (IGTP), 08916 Badalona, Catalonia, Spain; Tuberculosis Research Unit, Germans Trias i Pujol Research Institute (IGTP), Badalona, Catalonia, Spain; Genetics and Microbiology Department, Universitat Autònoma de Barcelona, Bellaterra, Catalonia, Spain; Tuberculosis Research Unit, Germans Trias i Pujol Research Institute (IGTP), Badalona, Catalonia, Spain; Comparative Medicine and Bioimage Centre of Catalonia (CMCiB), Germans Trias i Pujol Research Institute (IGTP), 08916 Badalona, Catalonia, Spain; Microbiology Department, Laboratori Clínic Metropolitana Nord, Germans Trias i Pujol University Hospital, 08916 Badalona, Catalonia, Spain; Tuberculosis Research Unit, Germans Trias i Pujol Research Institute (IGTP), Badalona, Catalonia, Spain; Department of Veterinary Paraclinical Studies, University of Gondar, Gondar, Ethiopia; Tuberculosis Research Unit, Germans Trias i Pujol Research Institute (IGTP), Badalona, Catalonia, Spain; Comparative Medicine and Bioimage Centre of Catalonia (CMCiB), Germans Trias i Pujol Research Institute (IGTP), 08916 Badalona, Catalonia, Spain; Microbiology Department, Laboratori Clínic Metropolitana Nord, Germans Trias i Pujol University Hospital, 08916 Badalona, Catalonia, Spain; Genetics and Microbiology Department, Universitat Autònoma de Barcelona, Bellaterra, Catalonia, Spain; Centro de Investigación Biomédica en Red en Enfermedades Respiratorias (CIBERES), Instituto de Salud Carlos III (ISCIII), Madrid, Spain

**Keywords:** Drosophila melanogaster, immunity, sexual dimorphism, reproductive status, mycobacterial infections, Mycobacterium marinum

## Abstract

Sex and reproductive status of the host have a major impact on the immune response against infection. Our aim was to understand their impact on host tolerance or resistance in the systemic *Mycobacterium marinum* infection of *Drosophila melanogaster*. We measured host survival and bacillary load at time of death, as well as expression by quantitative real-time polymerase chain reaction of immune genes (diptericin and drosomycin). We also assessed the impact of metabolic and hormonal regulation in the protection against infection by measuring expression of upd3, impl2 and ecR. Our data showed increased resistance in actively mating flies and in mated females, while reducing their tolerance to infection. Data suggests that Toll and immune deficiency (Imd) pathways determine tolerance and resistance, respectively, while higher basal levels of ecR favours the stimulation of the Imd pathway. A dual role has been found for upd3 expression, linked to increased/decreased mycobacterial load at the beginning and later in infection, respectively. Finally, impl2 expression has been related to increased resistance in non-actively mating males. These results allow further assessment on the differences between sexes and highlights the role of the reproductive status in *D. melanogaster* to face infections, demonstrating their importance to determine resistance and tolerance against *M. marinum* infection.

## 1. Introduction

Reproduction and immunity are resource-intensive traits in their deployment and maintenance, thus, the existence of a trade-off between the two biological processes has been widely questioned in both mammals and non-mammals, especially when the resources are limited [1–5]. Its study is important to shed light on the impact of directional selection and evolution in the maintenance or the variation of these traits, thus helping to better characterize the relationship between these two physiological mechanisms [6].

In this regard, *Drosophila melanogaster* is a powerful model organism to study the interaction between reproduction and immunity [7]. Sexual dimorphism as well as effects in mating and reproduction have been extensively reported in *D. melanogaster* studies [8–10]. This, in addition to its well-characterized immune system [11] and to the great benefits in terms of cost, time-consumption and genetic tools availability [12], make *D. melanogaster* a good model to study a possible trade-off between the two physiological aspects. Immunity in *D. melanogaster* relies solely on innate immune response. Nevertheless, as in mammals, this immune response is also divided into humoral and cellular arms, and have Nuclear factor kappa B(NF-κB) family transcription factors and signal transduction pathways [11]. The humoral response is mainly based on the synthesis of antimicrobial peptides (AMPs) in the fat body of the fly regulated by two signalling pathways: the Toll and the immune deficiency (Imd) pathways, which induce the production of Drosomycin and Diptericin (as the main AMP of each pathway), respectively [13, 14]. Flies also have a simpler but complete Janus kinase/signal transducers and activators of transcription (JAK/STAT) pathway [15, 16]. Regarding cellular immunity in *Drosophila,* it is mediated by haemocytes. When a septic injury, tissue damage, or exposure to lipopolysaccharides occurs, haemocytes trigger the expression of Unpaired (Upd) 3, which activates the JAK/STAT pathway in the fat body and contributes to the humoral response activation and the upregulation of immune genes [15, 17].

It is well known that hormones and metabolism, as well as circadian cycles, have central roles in the regulation of systemic physiology, including immunity, in both insects and mammals [18–20]. The metabolic homeostasis in insects is mainly regulated by the insulin/insulin-like signalling (IIS) pathway. When there is an immune stimulus, the immune pathways interact with the IIS pathway in the fat body resulting in loss of energy storage and suppression of host growth [21–23]. This fact has been recently described as the ‘selfish immune system theory’, which is based on the fact that activated phagocytes release signalling molecules (selfish immune factors, SIFs) that regulate host energy to steal resources from other non-immune tissues [24–27]. Authors propose the insulin/insulin-like growth factor (IGF) antagonist Imaginal morphogenesis protein late 2 (Impl2)—which has been identified as a cancer-derived cachectic factor in flies—and Upd3 as potential SIFs in *Drosophila* [28–30].

Hormones also play an important role in the regulation of immunity [18]. In both mammals and insects it has been described that a hormonal-driven sexual dimorphism might exist affecting immunity (survival, pathology, bacterial load and activity in response to infections) at basal conditions, after a pathogenic challenge, and even upon ageing [31, 32]. Specifically in *D. melanogaster*, some studies on the subject revealed that these differences might be mediated by different immune players depending on the pathogen and the route of infection. For example, males appeared to be more resistant to systemic infections by *Providencia* species, and *Enterococcus faecalis*, while females seemed to be more resistant to *Pseudomonas aeruginosa*, *Staphylococcus aureus* and *Serratia* infections [9, 33, 34]. Recently, a role for some Toll receptors in mediating sex dimorphisms has arisen with the finding that the loss of Toll-7 reduces resistance to *P. aeruginosa* and *E. faecalis* in males but not in females in the Toll-7^g1–5^/Cyo mutant line in comparison with WT w^1118^ flies [35], although these results could be influenced by the different genetic background of the compared strains. In mammals, steroid hormones have been linked with these sex differences, and interactions between these type of hormones and the innate immune system of *Drosophila* have also been described [36, 37]. The steroid hormone Ecdysone is the main regulator of the insect life cycle [38]. In adult flies, Ecdysone is produced in the ovaries after mating, thus showing higher levels in females than in males [39–41]. Its signalling through the receptor complex formed by the Ecdysone receptor (EcR) and Ultrapiracle (Usp) is required for the proper expression of the pathogen-sensing receptor peptidoglycan recognition protein LC(PGRP-LC) and, thus, the production of Imd-dependent AMPs [42] and the cellular immunity [37, 43]. At the same time, Ecdysone levels are regulated by stress signals and it has been related to age-related immune changes, as its depletion increases *Drosophila’s* lifespan [44, 45].

It is not only sex that affects immunity, but also reproductive status. In *Drosophila*, mated females showed decreased survival, higher pathogen loads and reduced AMPs production after pathogenic infections [46–48], which did not occur when the agent was non-pathogenic [4, 49, 50]. However, when mated female flies are unable to generate eggs, they do not have this reduction in immunity after mating [48]. This makes evident the trade-off between the reproductive factor of producing eggs and immunity due to reproduction costs. On the other side, immunity has also shown to take a reproductive toll by reducing egg viability in mated females [51]. However, if females were fed yeast *ad libitum*, both fecundity and resistance to infection were improved, suggesting, altogether, that reproduction and immunity compete for energy resources [48, 51, 52]. Along the same lines, males exposed to a higher number of females also showed increased susceptibility to bacterial infections [53]. Conversely, it has been also demonstrated that, despite the significant metabolic cost of infection, in infected wild-type Canton S (CS) mated male and female flies the pre-copulatory reproductive behaviours are preserved [7, 54]. Likewise, mated males have shown better survival curves against *Pseudomonas entomophila* infection as well as a better bacterial clearance ability against *Providencia rettgeri* than virgin ones, showing absence of reproduction-immunity trade-off [6].

In humans, sexual dimorphism has been also observed in the pathogenesis of some infectious diseases such as tuberculosis [55] which has been the leading cause of death due to a single pathogen (*Mycobacterium tuberculosis*, Mtb), surpassing the second deadliest bacterial infection: the plague (caused by *Yersinia pestis*). In the last years, it has only being exceeded by the viral COVID-19 pandemic on 2020 [56]. *Mycobacterium marinum* is the most common pathogenic mycobacteria used for studying host-pathogen interactions in non-mammal animal models because it is a natural pathogen of ectotherms causing a granulomatous infection that highly resembles tuberculosi (TB) in humans [57–59]. The infection of this mycobacterium in the *D. melanogaster* model has also been well characterized. The microorganism is able to kill flies even with very low initial doses, at the early stages of the infection, as it is able to replicate inside *Drosophila* haemocytes [60]. It has also been described that the formation of lipid droplets inside phagocytic cells derived from the production of Upd3 in these cells benefits the intracellular growth of the pathogen, thus resembling the formation of foamy macrophages in humans. As the infection evolves, *M. marinum* causes a progressive loss in energy storage inducing a cachexia-like process that eventually, accompanied by widespread tissue damage, is responsible for killing the flies. This wasting process is mediated by the disruption of the IIS pathway [61].

Some studies have addressed the possibility of a trade-off between reproduction and immunity in *D. melanogaster* analysing how the interaction between sex of the fly (male, female), its reproductive status (virgin and mated kept alone or together) and a specific infection affect the immunity of the fly [8–10]. However, this is the first study that does it with mycobacteria, specifically with the model that is currently used in *Drosophila* to study tuberculosis. In this study, we have assessed the role of sexual dimorphism and reproductive status on the response to the infection by *M. marinum* in *D. melanogaster*. We have also assessed the differences that these features have on the innate immune response triggered by the pathogen by measuring the expression of Toll- and Imd-dependent AMPs, *drosomycin* and *diptericin*, respectively. In addition, due to the importance that metabolism has on the *M. marinum* infection in flies [61], we have assessed the expression levels after the infection of *upd3* and *impl2* based on previous studies that described the role that these molecules play in the metabolic regulation of the host during infections [25, 62]. While doing this, we reinforced the fact that mycobacterial infections, such as tuberculosis, should be tackled considering all these parameters.

## 2. Materials and methods

### 2.1. Fly stocks and husbandry

Oregon-R-C wild-type flies were obtained from the Bloomington Drosophila Stock Centre (Indiana University) and they belong to stock number #5. Flies were raised on a standard cornmeal medium (Nutri-Fly^®^ Bloomington Formulation, Genesee Scientific) prepared following manufacturer’s instructions and maintained at 25°C, 65–70% humidity with a 12 h light/dark cycle. Male and female flies were aged for 3–5 days post-eclosion before experimentation.

### 2.2. Mycobacterial stock preparation and infection

*Mycobacterium marinum* E11 strain resistant to kanamycin (a kind gift from Wilbert Bitter, Vrije Universiteit Amsterdam) was used [63]. The mycobacterial strain was cultured in 7H9 complete medium complemented with 20 µg/ml of antibiotic and placed at 30°C with constant agitation (170 rpm) for 10 days (until an OD_600nm_ of 1.5). The cultures were centrifuged for 5 min at 4000g, resuspended in phosphate-buffered saline (PBS) with 0.2% Tween 80 and centrifuged again for 5 min at 500g to remove clumps. Supernatants were transferred to a new tub, centrifuged for 5 min at 4000g and resuspended in 1ml of 7H9 with 15% glycerol. Mycobacterial cultures were then aliquoted and frozen at −80°C. Each stock was tittered after being frozen at least overnight.

For systemic infections, an aliquot was defrosted and centrifuged at 15 000g for 5 min. Pellet was rinsed twice with sterile PBS and diluted to the proper concentration. For the mycobacterial infection, inoculation doses of 50, 500, 5000, 50 000 colony forming units(CFUs) per fly were used. Flies injected with sterile PBS were used as uninfected control. Fourteen nanolitres of each solution were injected systemically employing a nano-injector (Nanoject II, Drummond) into the abdomen of anaesthetized flies.

### 2.3. Experimental design

Flies were divided into three experimental groups: virgin flies, mated flies that were separated by sex after the infection and flies that were allowed to mate throughout the infection (actively mating). Virgin flies were collected at the 2–3 h post-eclosion, and kept in same-sex groups before and after experimentation. In all experiments, male and female flies were kept in groups of 30 individuals, either only one sex or equally distributed between males and females, and were all infected 3–5 days post-eclosion. Survival, bacterial load upon death (BLUD) and gene expression were measured for each experimental group.

Overall, the experiment consisted of 30 male and 30 female flies for each experimental group (each reproductive status) to track survival and BLUD. Flies’ survival was tracked for a month, as all infected flies at all inoculation doses were already dead at least 1 week or 10 days before. Dead flies were collected twice a day, washed with 70% ethanol, and rinsed twice with PBS. Each fly was then mechanically homogenized into 200 μl of sterile PBS, diluted and plated into 7H10 plates complemented with kanamycin, and incubated for 10 days at 30°C. We performed all experiments in triplicate with a total of 90 males and 90 females per group. In the first replicate, we assessed for differences between flies collected first in the morning (8 am) and in the afternoon (5 pm). No statistically significant differences were observed among bacillary loads depending on the time dead flies were collected (Supplementary Fig. 1). For gene expression analysis, additional males and females for each condition were infected and three pools of three flies were analysed per each group and time point. Experiments were also performed in triplicate.

### 2.4. Tolerance and resistance

Previous studies have defined three defensive strategies against infections: qualitative resistance, as the ability to remain uninfected upon pathogen exposure; quantitative resistance, as the ability to reduce the pathogen load; and tolerance, as the ability of the host to maintain the fitness, measured by the survival to the infection in our study, given a certain pathogen load [64, 65]. Given our model of study in which the minimum lethal dose of the pathogen is 1, meaning that a single mycobacterium is able to kill the host, we did not assess qualitative resistance but focussed on tolerance and quantitative resistance.

Tolerance was defined by the slope of the regression between the host fitness against the pathogen burden [65–70], with more tolerant groups presenting less steep slopes. We used flies’ survival rates as an indicator of host fitness and inoculation dose as the pathogen burden, because it highly correlates with both survival times and BLUD. Uninfected flies injected with sterile PBS were included in the analysis to account for the fitness of each group in the absence of infection, commonly referred to as general vigour [65]. On the other hand, resistance was measured as the Y-intercept of the regression line between the BLUD and the inoculation dose. When slopes are equal, the lower the Y-intercept the more resistant the group is [65, 67, 68].

### 2.5. Gene expression by quantitative real-time PCR

The differential gene expression was assessed at 24 h, 5 days and 10 days post-infection. Overall, a total of nine pools of three flies infected with 500 CFUs each were analysed per sex, group and time point, obtained from the three independent experiments. All flies were preserved in RNA later (Fisher Scientific, S.L.) and stored at −80°C before RNA extraction. Total RNA was extracted from flies using the MasterPure™ Complete DNA and RNA Purification Kit (Lucigen) and 600 μg of RNA were converted to cDNA with the PrimeScript RT Master Mix (Takara), following the instructions of the kit’s manufacturers for both procedures. cDNA was stored at −20°C until used.

Quantitative real-time polymerase chain reaction (rt-qPCR) was carried out using the KAPA SYBR® FAST Mix (Sigma) on a LightCycler 480 (Roche Diagnostics) system. RT-qPCR conditions were 95°C for 5min followed by 40 cycles of 95°C for 10 s and 60°C or 62°C for 20 s. The specificity of each pair of primers was checked by melting curve analysis (95°C for 5 s, 65°C for 1 min and a continuous rise in temperature to 97°C at 2.5°C/s ramp rate followed by 97°C for 30 s ). To check reproducibility, each assay was performed with technical triplicates for each biological sample. The relative transcripts levels of target genes were calculated using the 2^-ΔΔCT^ method [71] with r*pl32* used as the reference gene for normalization of target gene abundance. The oligonucleotides used are detailed in Table 1.

**Table 1.** Oligonucleotide sequences used for real-time PCR

Gene	Primer forward (5ʹ–3ʹ)	Primer reverse (5ʹ–3ʹ)
Rpl32	ACAGGCCCAAGATCGTGAAG	TCGACAATCTCCTTGCGCTT
Diptericin	GGCTTATCCGATGCCCGACG	TCTGTAGGTGTAGGTGCTTCC
Drosomycin	CCAAGCTCCGTGAGAACCTT	CAGGTCTCGTTGTCCCAGAC
Upd3	GCAAGAAACGCCAAAGGA	CTTGTCCGCATTGGTGGT
Impl2	GCCGATACCTTCGTGTATCC	TTTCCGTCGTCAATCCAATAG
Ecdysone receptor	CAACAGCTCGGACTCAATATTCTT	GTTCTCCTCCTGGGTAATCTGAA

### 2.6. Statistical analysis

Tolerance and resistance analyses were carried out in GraphPad Prism (version 9.0.0). Survival, CFU counts and gene expression data were checked for outliers with the Robust regression and Outlier removal(ROUT) method (Q = 1%) and for normality with the Shapiro–Wilk normality test. For multiple comparisons we performed Tukey’s test for normally distributed data and Dunn’s test for non-normally distributed data. For comparisons between two groups we performed unpaired t test for normally distributed data and Mann–Whitney test for non-normally distributed data. A linear regression model was used to analyse the tolerance and resistance. Slopes and Y-intercepts were analysed for statistically significant differences with the Extra sum-of-squares F test. The principal component analysis (PCA) was performed with the Factoextra package in R (version 4.0.1). We have analysed each replicate separately to ensure the consistency of the outcomes prior to analyse them together.

## 3. Results

### 3.1. The effect of reproduction in tolerance and resistance to *M. marinum* infections is sex-dependent and reproductive status-dependent

To study tolerance and resistance to the infection by *M. marinum*, flies were injected systemically with increasing doses of the pathogen (50, 500, 5000 and 50 000 cfu/fly) or with PBS as control, and kept at 25°C throughout the experiment.

**Figure 1.**
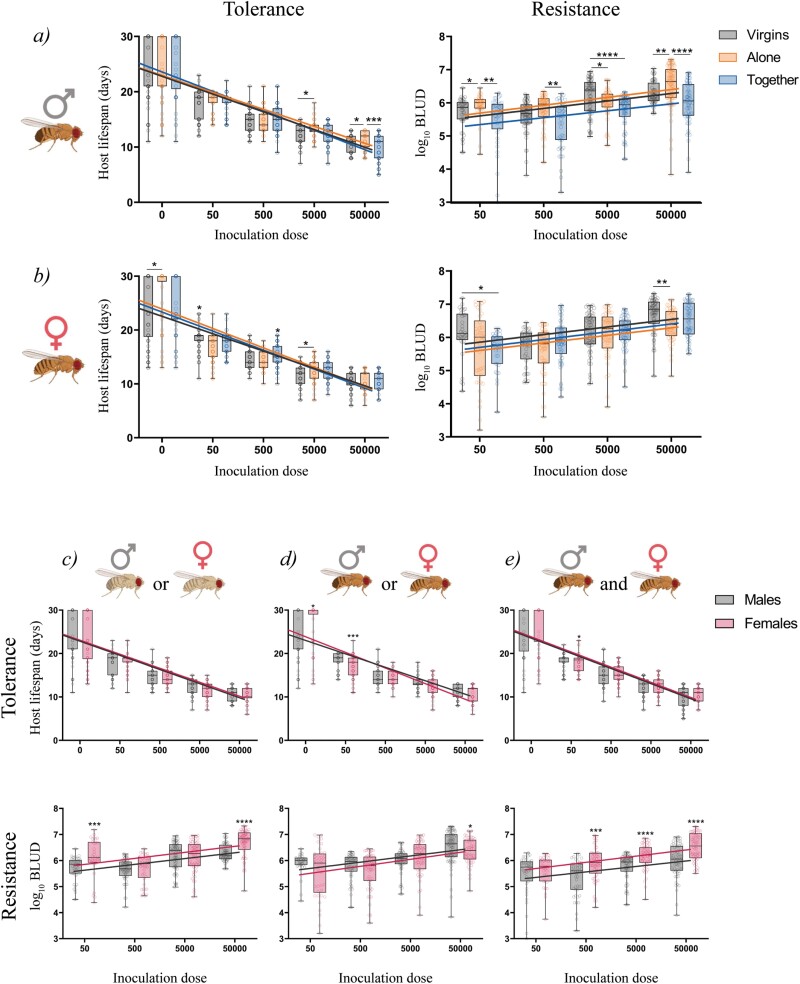
Tolerance and resistance of *D. melanogaster* to *M. marinum* infections depending on the reproductive status or the sex of the flies. (a) Males that were not in presence of females (virgins and alone) were more tolerant but less resistant than those kept together with females. (b) In females, virgins were more tolerant but less resistant than mated females, independently of the presence or absence of males. When comparing both sexes for each reproductive status, we observed that males were more resistant when virgins or together with females, although no differences in tolerance were found in these groups (c and e). When flies were kept alone after infection, females showed higher general vigour and resistance, but lower tolerance to infection (d). Lines represent the regression lines fitted for each group and each circle represents an individual. Both survival and bacillary load between the groups were analysed independently for each inoculation dose and were tested for normality. Statistically significant differences were represented as follow: *P ≤ 0.05, **P ≤ 0.01, ***P ≤ 0.001, and ****P ≤ 0.0001 (Kruskal–Wallis test).

Our study revealed no significant differences in the general vigour (understood as the survival of non-infected flies for each group) in males, independently of their reproductive status. Instead, when looking to results of infection, they showed that those males kept together with females were less tolerant to the infection by *M. marinum* compared to males kept alone and virgin males (*P* = 0.0026; *P* = 0.0447, respectively), but more resistant (*P* < 0.0001) (Fig. 1a; Table 2). Altogether results suggest that males reproductively active (males kept together) are also more immune activated in response to infection, rather than those having previously being mated or not.

**Table 2.** Analysis of tolerance and resistance to *M. marinum* infections depending on the reproductive status

Males			
Tolerance			
Group	Slope	Comparison	*P* value
Virgins	−3.149	Alone	0.3492
Alone	−3.014	Together	0.0026**
Together	−3.447	Virgins	0.0447*

Resistance			
Group	Y-inter	Comparison	*P* value
Virgins	5.138	Alone	0.0325*
Alone	5.196	Together	<0.0001****
Together	4.913	Virgins	<0.0001****

Females			
Tolerance			
Group	Slope	Comparison	*P* value
Virgins	−3.165	Alone	0.0175*
Alone	−3.536	Together	0.6273
Together	−3.465	Virgins	0.0484*

Resistance			
Group	Y-inter	Comparison	*P* value
Virgins	5.366	Alone	<0.0001****
Alone	4.947	Together	0.2354
Together	5.100	Virgins	0.0067*

Significance (*P* < 0.05) is indicated by asterisks. Differences in resistance’s slopes were not statistically significant between groups.

On the other hand, the general vigour of PBS-injected virgin females was significantly lower when compared with both mated groups. In fact, if we look to females’ response to infection, we saw that virgin females were more tolerant (*P* = 0.0175; *P* = 0.0484) but less resistant (*P* < 0.0001; *P* = 0.0067) to the infection, compared to both mated groups (mated kept alone and mated kept together, respectively) (Fig. 1b; Table 2). Contrary to males, these results suggested that the immune phenotype to the infection in female flies was more conditioned by whether they have been mated, regardless of whether they were reproductively active at the time of infection or not.

When we compared tolerance and resistance of males and females for each reproductive status independently, data showed that virgin flies differed neither in general vigour nor in overall tolerance to the infection, but males were significantly more resistant (*P* < 0.0001) (Fig. 1c; Table 3). The same pattern was observed in flies that were kept together in equal proportions throughout the procedure: both males and females had the same general vigour and the same tolerance levels, but males showed up to be more resistant (*P* < 0.0001) (Fig. 1e; Table 3). On the contrary, when flies had mated but were kept separated by sex after the infection, females showed increased general vigour and resistance (*P* = 0.0288), but reduced tolerance to the infection with *M. marinum* compared with males (*P* = 0.0008) (Fig. 1d; Table 3).

**Table 3.** Analysis of tolerance and resistance to *M. marinum* infections depending on the sex

Tolerance			
Group	Slope males	Slope females	*P* value
Virgins	−3.171	−3.142	>0.9999
Alone	−3.014	−3.505	0.0008***
Together	−3.447	−3.351	0.5099

Resistance			
Group	Y-inter. males	Y-inter. females	*P* value
Virgins	5.138	5.366	<0.0001****
Alone	5.196	4.947	0.0288*
Together	4.913	5.196	<0.0001****

Significance (*P* < 0.05) is indicated by asterisks. Differences in resistance’s slopes were not statistically significant between groups.

All these results allow us to classify from tolerant or resistant to the *M. marinum* infection each fly according to its sex and each reproductive status. Being more tolerant and less resistant: virgin males, mated males kept alone and virgin females. And more resistant and less tolerant: mated and kept alone males and females, and females kept together.

### 3.2. The humoral innate immune response against *M. marinum* infection depends on the reproductive status of the host

**Figure 2.**
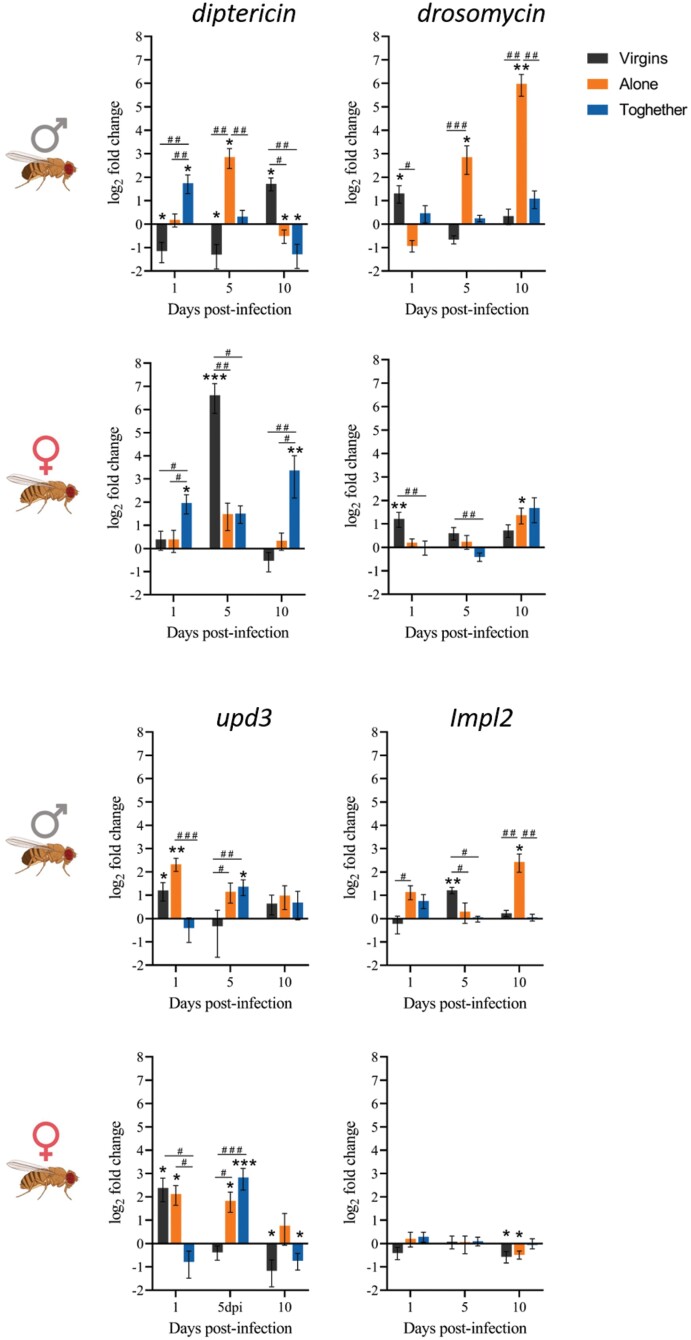
Expression of innate immunity (a) and metabolic (b) genes during the infection in males and females depending on their reproductive status. Gene expression relative to the internal control gene rpl32 was quantified in nine replicate pools of three males or females exposed to the infection with *M. marinum* relative to their expression in uninfected controls. Each group at each time point was compared to its relative control independently (the line in each graph represents the controls’ relative expression with a log_2_ fold change of 0). Data was analysed for normality and significant differences were represented as follow: *P ≤ 0.05, **P ≤ 0.01 (Welch’s corrections for normally distributed data and Mann–Whitney test for not normally distributed data). Differences among the different groups were also analysed for each time point independently following the same approach. Differences were represented as follow: ^#^*P* ≤ 0.05, ^##^*P* ≤ 0.01, ^###^*P* ≤ 0.001, and ^####^*P* ≤ 0.0001.

The differences in the innate immune response triggered by the infection in each reproductive status were measured by the expression of the antimicrobial peptides Diptericin and Drosomycin in flies infected with 500 CFU of *M. marinum* at different time points (Fig. 2a). These AMPs are regulated by the Imd and the Toll pathways, respectively. In males, only those kept together with females showed immediate significant production of Diptericin, while the other groups showed a delayed production of this AMP. Moreover, virgin males presented significantly increased production of Drosomycin immediately after the infection, while in males alone this production was delayed, and no significant changes were observed in males kept together with females. In females, we observed similar expression profiles as males for each reproductive status: females kept together with males had immediate production of Diptericin, but not Drosomycin; virgin females had delayed production of Diptericin, but early production of Drosomycin; females alone showed delayed or null production of both AMPs.

These results for the expression levels showed a link with the phenotypic results obtained previously. We found that the activation of the Toll pathway (production of Drosomycin) is associated with more tolerant but less resistant phenotypes in both males (virgins and mated kept alone) and females (virgins). In addition, the more sustained this activation was (mated males kept alone) the lower resistance was observed. On the other hand, we also observed that while early activation of the Imd pathway was associated with the more resistant groups (males and females together), late or null activation of this pathway seemed to be linked with reduced resistance with the exception of females alone. These interactions were also observed when comparing the expression levels between males and females for each reproductive status (Supplementary Fig. 2a). We found that the less resistant groups (virgin females, females kept together with males, and males kept alone) showed significantly increased production of both AMPs late in the infection, while the more tolerant group (males kept alone) had increased production of Drosomycin. Finally, results also suggest that females kept alone after mating may acquire their resistance through a mechanism that does not involve the humoral innate immunity, as they did not show any activation of the Imd pathway throughout the infection.

### 3.3. The metabolic regulation during infection is altered by the reproductive status of the host

The link with the metabolism, especially with the IIS pathway, together with the suggested role for Upd3 and Impl2 as ‘SIFs’, prompted us to investigate whether a differential expression of these molecules within the different reproductive status of the host might also influence the tolerance and resistance levels to the infection by *M. marinum* (Fig. 2b).

In males, both virgins and those kept alone showed significant early overexpression of *upd3*, which was not sustained later in the infection. On the other hand, those males that were kept together with females during the whole procedure presented a significant overexpression of this gene later in the infection. In females, virgins showed a significant early but not sustained overexpression of *upd3*, while females that have mated presented a significantly increased expression of this gene later in the infection whether they were in the presence or absence of males. Overall, these results suggest a dual role for *upd3*, as its early expression correlated with more tolerant but less resistant groups while its expression later in the infection correlated with more resistant and less tolerant groups.

Finally, assessing the expression of *impl2* during the *M. marinum* infection we observed no significant differences among females depending on their reproductive status. On the other hand, both virgin males and mated males kept alone showed a significant increase in the expression of this gene later in the infection, but not sustained. These results suggested that, in males, the punctual expression of *impl2* might increase tolerance to the infection with *M. marinum*, but not in females.

We also compared the expression levels of both genes between males and females for each reproductive status independently (Supplementary Fig. 2b). We found that females have an overall increased expression of *upd3* compared to males, while *impl2* seemed to only play a role in males with its late production correlating with increased tolerance (males kept alone).

### 3.4. Induction of the ecdysone receptor after infection correlated with basal levels prior to the infection in males, but not in females

**Figure 3.**
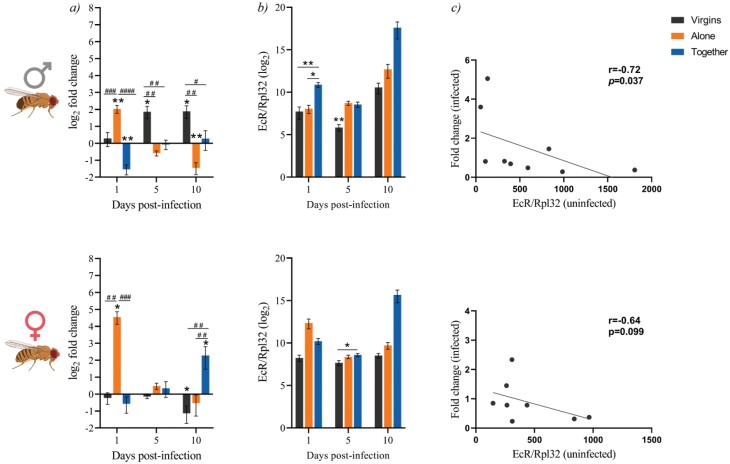
Expression of the ecdysone receptor (EcR) in males (up) and females (down) depending on their reproductive status after the infection (a). Gene expression relative to the internal control gene rpl32 was quantified in nine replicate pools of three males and three females each exposed to the infection with *M. marinum* relative to their expression in uninfected controls. Each group at each time point was compared to its relative control independently (the line in each graph represents the controls’ relative expression with a log_2_ fold change of 0). Data was analysed for normality and significant differences were represented as follow: *P ≤ 0.05, **P ≤ 0.01 (Welch’s corrections for normally distributed data and Mann–Whitney test for not normally distributed data). Differences among the different groups were also analysed for each time point independently following the same approach. Differences were represented as follow: ^#^*P* ≤ 0.05, ^##^*P* ≤ 0.01, ^###^*P* ≤ 0.001, and ^####^*P* ≤ 0.0001. Basal expression levels of the EcR gene in uninfected flies (b) were calculated using the 2^-ΔCT^ method with the rpl32 gene for normalization (all values were multiplied by 10^4^ for more visual results). Groups were compared independently for each time point. Data was analysed for normality and significant differences were represented as follow: *P ≤ 0.05, **P ≤ 0.01 (Kruskal–Wallis test). Finally, the correlation between the basal levels and the fold change after infection was performed using the nonparametric spearman correlation test (c).

We also evaluated the expression levels of the ecdysone receptor (EcR) due to the tight relationship of the Ecdysone pathway with immunity and reproduction. We measured the relative expression of this gene after the infection and the basal expression levels when injected with PBS for each reproductive status (Fig. 3c).

We found that virgin males significantly induced the expression of the receptor later in the infection, while males kept alone did it immediately after. On the other hand, when males were kept together with females, the infection triggered a significant repression of the receptor (Fig. 3a). We also found that males presented a significant correlation between the basal level of EcR and the induction of this receptor post-infection, in which lower basal levels translated into higher activation of this gene post-infection (Fig. 3b and c). In females, virgins showed no changes neither in the basal levels nor in the induction of EcR after the infection. Females that had mated showed similar patterns as their males’ counterparts, while females kept alone showed decreasing induction of EcR when infected, but no significant changes on the basal levels (Fig. 3a and b). However, the correlation between basal levels and induction of EcR after the infection was not statistically significant in females (Fig. 3c).

The results also showed a link between the basal expression levels of EcR and the resistance to the infection. Those groups with higher basal expression levels (males and females kept together and females alone) were more resistant to the infection, while those groups with lower basal levels (virgin flies and males alone) were less resistant.

When comparing both basal and induction levels between males and females (Supplementary Fig. 3a), we observed that virgin males presented lower basal levels but increased production of EcR after the infection, while flies separated after the infection showed no significant different basal levels but females increased its production after the infection. Finally, when flies were kept together throughout the procedure, no differences at basal levels among sexes were found, but a late induction in females.

### 3.5. Gene expression upon infection in *D. melanogaster* might be influenced by sexual dimorphism

**Figure 4.**
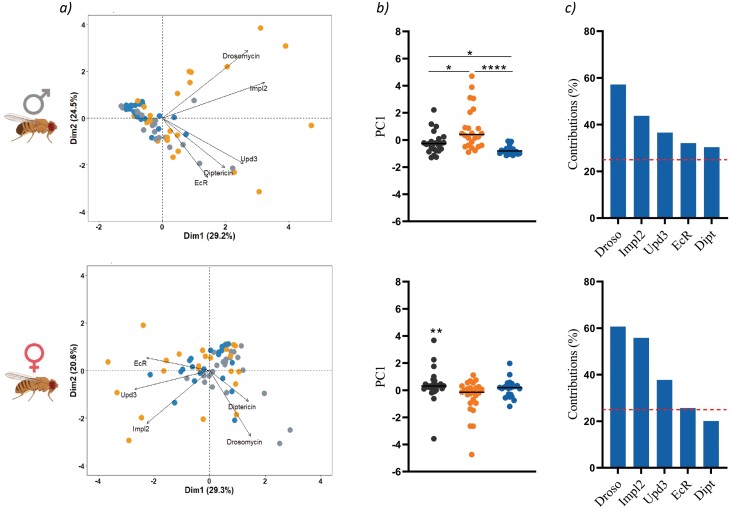
Heterogeneity of gene expression among flies with different reproductive status. PCA based on expression of genes of interest in males and females infected with *M. marinum* at all time points (a), PC1 scores (b) and variable contribution to PC1 and PC2 (c). Each circle represents an individual and lines are means. Statistically significant differences were represented as follow: *P ≤ 0.05, ****P ≤ 0.0001 (Kruskal–Wallis test).

Finally, we assessed the overall correlation between all the genes studied. The PCA (Fig. 4a) performed with the relative expression levels of infected flies including all time points for each condition revealed that, in males, all three groups presented significantly different gene expression profiles (Fig. 4b top), which linked with all three groups presenting different tolerance and resistance profiles as well. In females, only virgins presented a significantly different gene expression profile (Fig. 4b bottom), also linking with the phenotypic results. Finally, the analysis of the variable contribution (Fig. 4c) for dimension 1 (PC1) and 2 (PC2), revealed that, in both sexes, the differences among reproductive status were mainly driven by the differential expression of Drosomycin, Impl2 and Upd3. The differential expression of EcR and Diptericin, turned out to play a less significant role in both sexes, especially in females.

We performed the same analysis but taking into consideration each time point separately in order to identify the chronological importance of the expression of each gene (Supplementary Fig. 4). Results showed several common patterns between males and females. First, flies kept alone presented an expression profile significantly different from the other two groups at 24 h post-infection whereas virgins were those significantly different later in the infection. When observing which genes were relevant to differentiate between groups, we observed that Drosomycin was crucial at all time points whereas Diptericin was only significant later in the infection. In addition, we found that Upd3 was relevant at the beginning (24 h) and at late stages (10 days) of the infection, and EcR was crucial at the beginning of the infection but not 10 days post-infection. On the other hand, we also showed that Impl2 was more relevant among males than in females.

## 4. Discussion and conclusions

In this study, we have been able to decipher whether reproductive status of *D. melanogaster* has a cost in immunity against infection and whether those flies display a specific phenotype for each of these reproductive status group in terms of tolerance and resistance to that infection. Several laboratory studies have revealed a significant ‘cost of mating’ to *Drosophila* females in the form of reduced longevity [72–75]. However, other studies have previously reported contrary results in both wild-caught [75] and laboratory-reared [76] flies. As we have observed in our study, they also showed no cost of mating in mated females flies compared to virgins when uninfected. These discrepancies are mostly driven by both the genetic background and the environment. Aspects as dietary and/or seasonal husbandry, even within the same laboratory, can substantially affect the study of mating costs and sex differences [75, 76]. However, in our study all experiments were performed independently and per triplicate. We performed all infections and other manipulations of flies at the same time slot, to not disrupt their circadian rhythm or create new possible variables for the results obtained. In addition, the wild-type *D. melanogaster* Oregon-R-C, has demonstrated to have a genetic background that does not affect its fertility, on contrary to other *D. melanogaster* crosses [77, 78]. One hypothesis that might explain this phenomenon we observed in our study is that non-infected flies have been exposed to a sterile injection. This sterile wounding induces an immune response that triggers the melanization of the damaged tissue via activation of the phenoloxidase cascade [79], and the production of reactive oxygen species derived from melanization has been shown to correlate with reduced longevity in flies [80]. It has been described that the seminal fluids induce the production of Juvenile hormone in mated females, which reduces the production of phenoloxidase [36, 81, 82]. Thus, these differences in the survival of uninfected female flies might be explained by the reduction in the oxidative response after a sterile damage in mated females, which is not altered in mated males.

Regarding the response to infections, a vast majority of studies described that both males and females reduce their resistance to some infections after mating [48, 53, 83]. Yet, some others reported no changes or even an increase in the resistance to infections after mating [6, 49, 54, 84]. In contrast, data presented herein shows that the opposite happens in both sexes during infection with *M. marinum*. Males increase their resistance to the infection after mating when they are together with females, although at the expense of a reduced survival, while females increase their resistance to *M. marinum* infection after mating, independently of the presence or absence of males. These sex-dependant results suggest that the benefit of mating might depend on the time elapsed since the cohabitation in males, but not in females.

Overall, phenotypic results from this study show that the sexual dimorphism observed in *D. melanogaster* in the outcome of *M. marinum* infection is highly related to the reproductive status of the host. In addition, a negative correlation between tolerance and resistance has been shown in our study, probably because the energy required to reduce the bacillary load directly affects other physiological mechanisms and this results in reduced survival. Our results together with previous published studies seem to indicate that the existence of a trade-off between immunity and reproduction is specific for each host-pathogen system studied, and thus, when assessing the differences in the tolerance and/or resistance of *D. melanogaster* to other infections, the hosts’ reproductive situation needs to be characterized.

Regarding the hormonal levels and their relation with the immune response, our data show that certain EcR levels are required for the flies after the infection, as flies with higher basal levels presented lower induction or repression of the gene post-infection. However, this correlation was only statistically significant in males. Previous studies showed that ecdysone induces the expression of the receptor PGRP-LC and, thus, modulates the Imd pathway [42]. Other studies had related the Imd pathway in the control of resistance to infections [85], while its negative regulation mediates tolerance to infections [86], although any of these were performed in mycobacterial infections. Our data support these findings, as those flies with prior higher basal levels of EcR showed higher expression levels of Diptericin immediately after infection and correlate with the more resistant phenotypes, while flies that did not show early production of this AMP were more tolerant to the infection.

Several studies performed with a wide range of pathogens have described the Toll pathway as key in determining resistance to infections [35, 87, 88], and even to be essential for resistance in males, but not in females [9]. However, no role in increasing tolerance to infection has been described for this pathway previously. In our study, we showed that production of the Toll-dependant AMP, Drosomycin, early in the infection was linked with increased tolerance but reduced resistance to *M. marinum* infection in flies, while their activation in later stages of the infection correlated with increased resistance. Altogether, these data suggest a role for the Toll pathway in determining both tolerance and resistance to *M. marinum* infections. Recent studies have described a Toll-dependent metabolic switch that directs fatty acids from neutral cellular storage toward phospholipid biosynthesis [89], which might reduce the lipid droplet accumulation inside phagocytic cells and, thus, hindered the intracellular replication of *M. marinum*.

This study also suggests a dual role for Upd3 in *M. marinum* infections. Our data correlate with previous studies that linked Upd3-deficient flies with a reduction of lipid droplet accumulation within cells and reduced bacillary loads [62], showing that flies with increased expression of Upd3 early in the infection presented higher bacillary loads. Yet, this study also suggests a second role for Upd3, as its production late in the infection correlate with increased resistance, probably due to its role in increasing the metabolism of phagocytic cells [27]. In addition, our data also suggest that the increased resistance in females alone might be driven by Upd3 rather than by innate immune pathways. Finally, the expression of Impl2 during *M. marinum* infections seems to be only relevant in males that are not actively mating and to be related to increased bacillary loads, although further studies should be performed to determine its role during mycobacterial infections in *D. melanogaster*.

In this study, we have focussed on characterizing the phenotypic immune profile of a population of *D. melanogaster* individuals (differing by their reproductive status) in response to a *M. marinum* infection. Lastly, to be able to correlate these findings with their evolutionary implications (e.g. changes in egg investment or genetic changes among individuals in a population among generations) it would be necessary to study how *D. melanogaster* evolve to these traits (reproductive status selection regimens and infection to *M. marinum*) along several generations [6]. Only by doing this, we will be able to shed light, not only in the phenotypical but in the importance of this findings in the evolution of both species.

## 5. Concluding remarks

It has been extensively studied that males and females respond differently to the same infections. Many of these studies have focussed on establishing the physiological basis for these differences, but very few have studied how the reproductive status of the host affects males and females and the role it plays in the response to infection. This study aimed to show that the intrinsic differences observed between males and females during the infection by *M. marinum* were tightly related to the reproductive status of the host and that reproduction affects males and females differently. Here we show that being actively mating (kept together) increased the resistance but reduced the tolerance to the infection in males. The same phenotype was found in mated females regardless if they were kept alone or together after mating. All of this demonstrates that when assessing the sexual dimorphism of *D. melanogaster,* the hosts’ reproductive situation needs to be characterized.

The results obtained in this study also suggest a possible role for the Toll pathway in determining tolerance to infection, while it appears that the Imd pathway is associated with increased resistance. Results also showed a correlation between basal levels of EcR and its induction after the infection, suggesting that a certain amount of this receptor is required upon infection. This phenomenon was observed in both males and females in all reproductive statuses, although only in males was statistically significant. In addition, basal levels of EcR also correlated with higher expression levels of Diptericin.

Finally, we have also proposed a dual role for Upd3 upon infection with *M. marinum*. Our results show that an early production of this molecule correlate with higher bacillary loads. This might be linked with the Upd3-mediated lipid droplets accumulation inside phagocytic cells, which favours mycobacterial replication. However, the late production of this molecule is associated with lower bacillary loads, most likely due to its role in increasing phagocytic cells metabolism. Finally, we could only validate the role for Impl2 in increasing the tolerance in non-virgin non-actively mating males.

## Supplementary Material

eoad029_suppl_Supplementary_Figures_S1-S4

eoad029_suppl_Supplementary_Material
