# Supplementary material for: The reproductive status determines tolerance and resistance to Mycobacterium marinum in Drosophila melanogaster
Source: Evol Med Public Health. 2023 Sep 6;11(1):332–47. doi: 10.1093/emph/eoad029 (PMC10590161; doi:10.1093/emph/eoad029)
Supplement: eoad029_suppl_Supplementary_Figures_S1-S4 [file eoad029_suppl_supplementary_figures_s1-s4.docx]

The reproductive status of the host determines the tolerance and resistance to *Mycobacterium marinum* infection in *Drosophila melanogaster*

Marta Arch^1,2^, Maria Vidal^1,4^, Esther Fuentes^1-3^, Pere Joan Cardona^1-5*^

^1^ Tuberculosis Research Unit, Germans Trias i Pujol Research Institute (IGTP), Badalona, Catalonia, Spain.

^2^ Comparative Medicine and Bioimage Centre of Catalonia (CMCiB), Germans Trias i Pujol Research Institute (IGTP), 08916 Badalona, Catalonia, Spain.

^3^ Microbiology Department, Laboratori Clínic Metropolitana Nord, Germans Trias i Pujol University Hospital, Badalona, Catalonia, Spain

^4^ Genetics and Microbiology Department, Universitat Autònoma de Barcelona, Bellaterra,

Catalonia, Spain

^5^ Centro de Investigación Biomédica en Red en Enfermedades Respiratorias (CIBERES), Instituto de Salud Carlos III (ISCIII), Madrid, Spain

**SUPPLEMENTARY FILE**


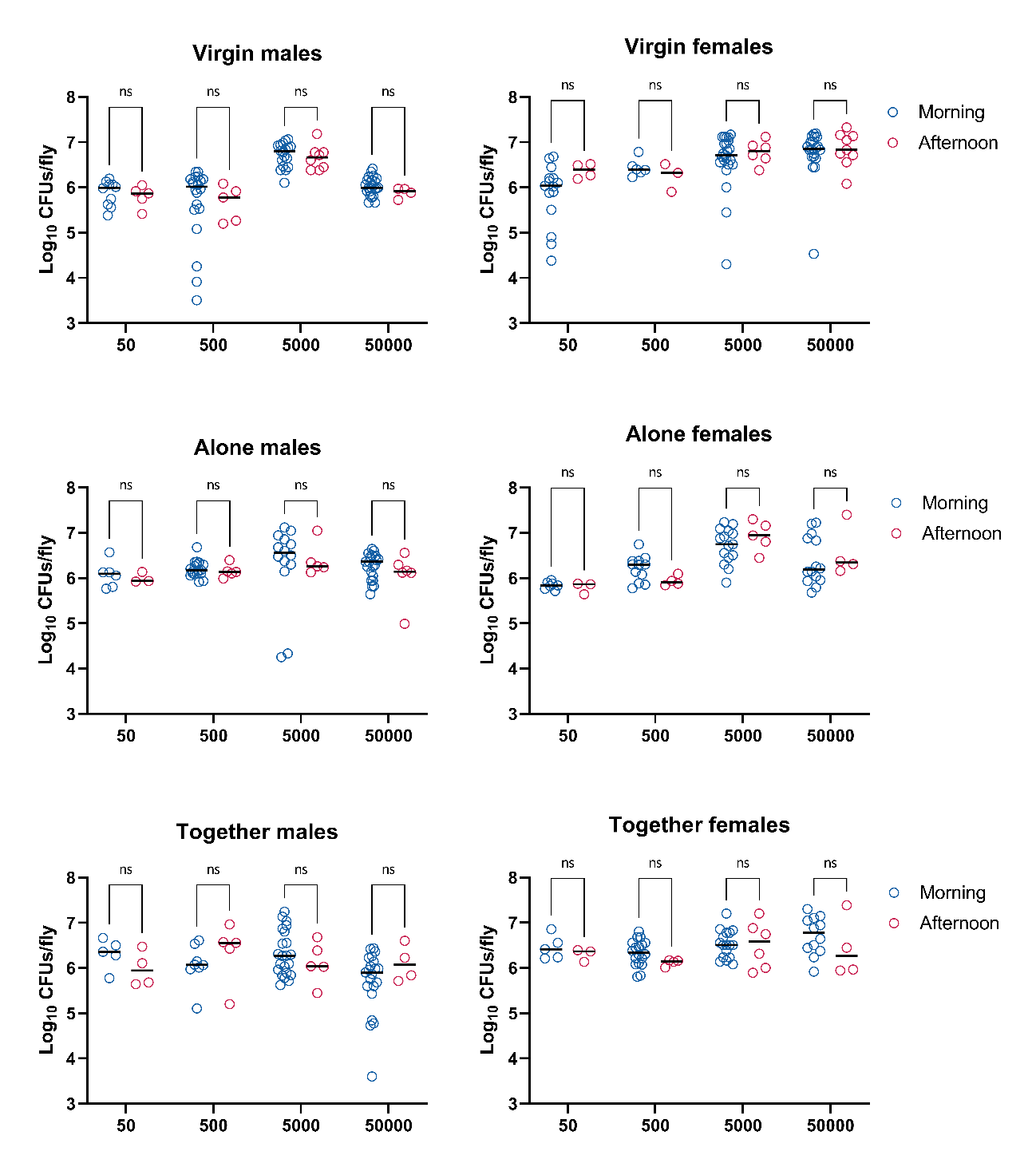


**Sup. Figure 1|** Comparison of the bacillary load upon death (BLUD) between flies collected in the morning (presumably several hours after death) and in the afternoon (recently dead). Each circle represents an individual. Bacillary load between the groups were analysed independently for each inoculation dose and were tested for normality. Statistically significant differences were represented as follow: *p≤0.05, **p≤0.01, ***p≤0.001, ****p≤0.0001 (Kruskal-Wallis test).


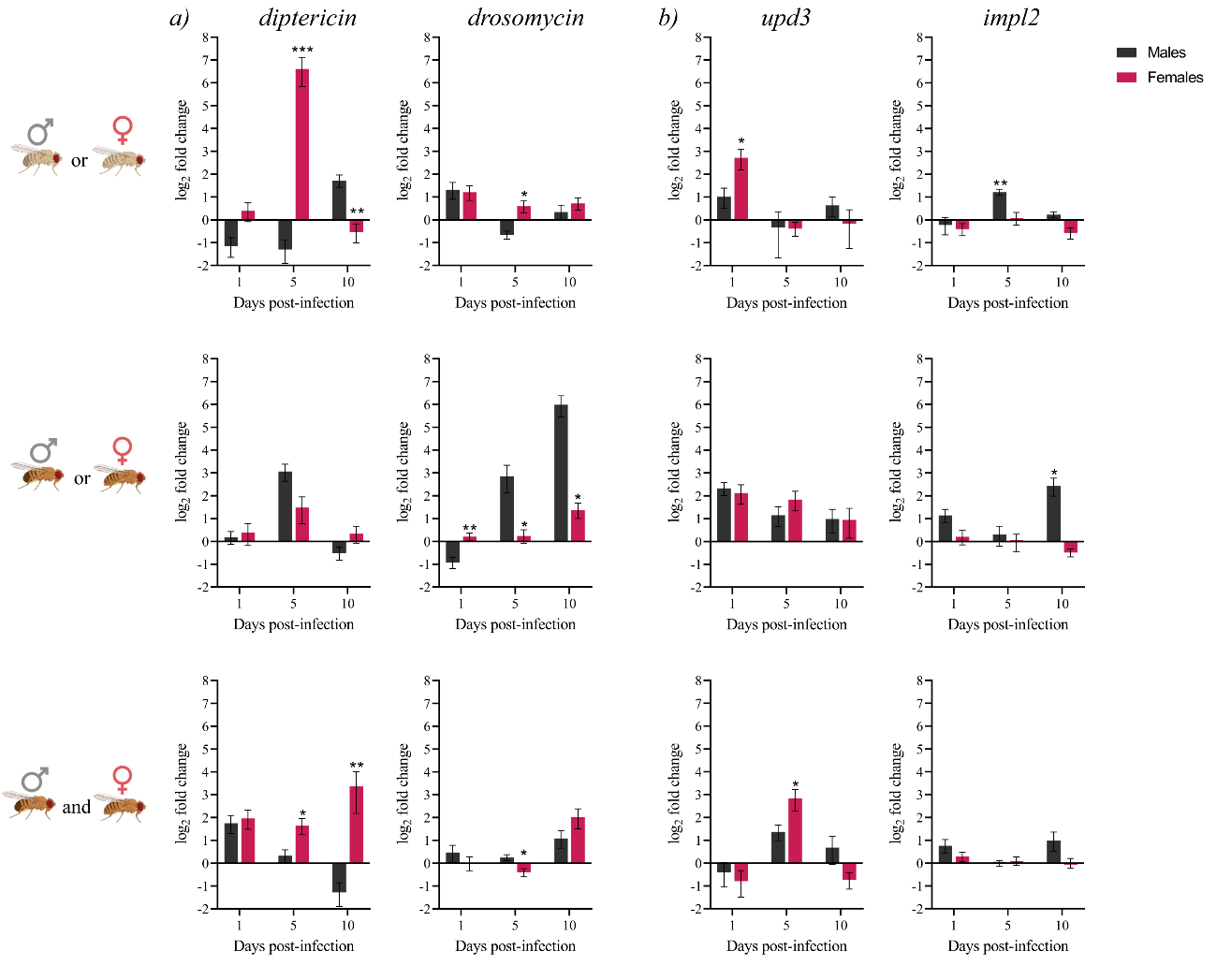


**Supplementary Figure 2|** Expression of innate immune (a) and metabolic (b) genes during the infection in flies depending on their sex for each reproductive status independently (from top to bottom: virgins, mated kept alone, and actively mating). Gene expression relative to the internal control gene rpl32 was quantified in 9 replicate pools of 3 males or females exposed to the infection with M. marinum relative to their expression in uninfected controls. Each time-point was compared independently (the line in each graph represents the controls’ relative expression with a log_2_ fold change of 0). Data was analysed for normality and significant differences were represented as follow: *p≤0.05, **p≤0.01, ***≤0.001 (Welch’s corrections for normally distributed data and Mann-Whitney test for not normally distributed data).


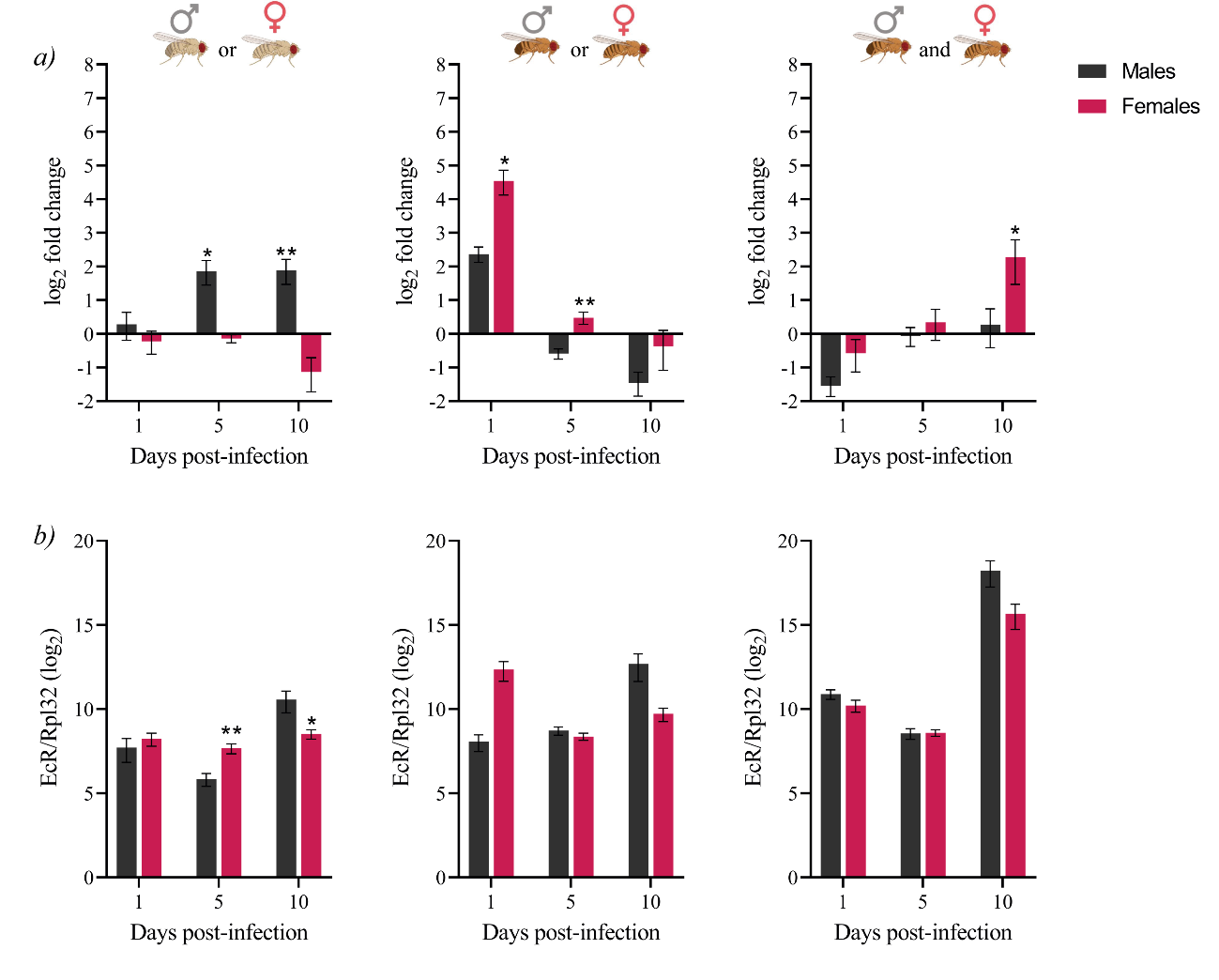


**Supplementary Figure 3|** Expression of the ecdysone receptor (EcR) in flies depending on their sex for each reproductive status independently (from left to right: virgins, mated kept alone, and actively mating). **(a).** Gene expression relative to the internal control gene rpl32 was quantified in 9 replicate pools of 3 males and 3 females each exposed to the infection with M. marinum relative to their expression in uninfected controls. Each time-point was compared independently (the line in each graph represents the controls’ relative expression with a log_2_ fold change of 0). Data was analysed for normality and significant differences were represented as follow: *p≤0.05, **p≤0.01 (Welch’s corrections for normally distributed data and Mann-Whitney test for not normally distributed data). Basal expression levels of the EcR gene in uninfected flies **(b)** were calculated using the 2^-ΔCT^ method with the rpl32 gene for normalization (all values were multiplied by 10^4^ for more visual results). Groups were compared independently for each time-point. Data was analysed for normality and significant differences were represented as follow: *p≤0.05, **p≤0.01 (Kruskal-Wallis test).


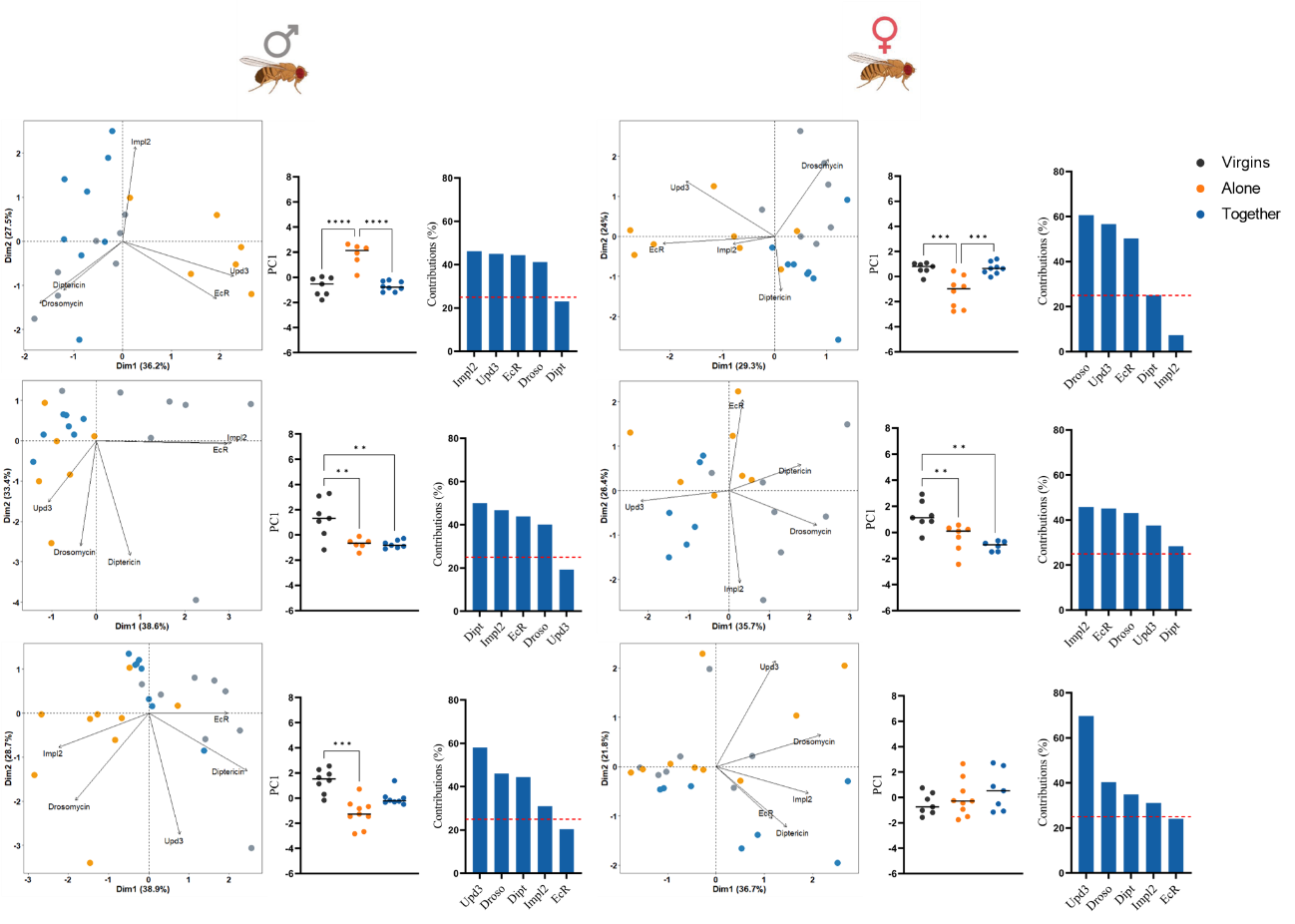


**Supplementary Figure 4|** Heterogeneity of gene expression among male and female flies with different reproductive status in each time point (from top to bottom: 24h, 5d and 10d post-infection). Principal component analysis (PCA) based on expression of genes of interest in males and females infected with M. marinum at each time-points (left), PC scores (middle) and variable contribution to PC1 and PC2 (right). Each circle represents an individual and lines are means. Statistically significant differences were represented as follow: *p≤0.05, ****p≤0.0001 (Tukey’s test for normally distributed data and Dunn’s test for non-normally distributed data).
